# Normalization of Ventilation Data from 4D-CT to Facilitate Comparison between Datasets Acquired at Different Times

**DOI:** 10.1371/journal.pone.0084083

**Published:** 2013-12-17

**Authors:** Kujtim Latifi, Vladimir Feygelman, Eduardo G. Moros, Thomas J. Dilling, Craig W. Stevens, Geoffrey G. Zhang

**Affiliations:** Department of Radiation Oncology, Moffitt Cancer Center, Tampa, Florida, United States of America; University of California San Francisco, United States of America

## Abstract

**Purpose:**

The 4D-CT data used for comparing a patient’s ventilation distributions before and after lung radiotherapy are acquired at different times. As a result, an additional variable – the tidal volume (TV) – can alter the results. Therefore, in this paper we propose to normalize the ventilation to the same TV to eliminate that uncertainty.

**Methods:**

Absolute ventilation (AV) data were generated for 6 stereotactic body radiation therapy (SBRT) cases before and after treatment, using the direct geometric algorithm and diffeomorphic morphons deformable image registration (DIR). Each pair of AV distributions was converted to TV-normalized, percentile ventilation (PV) and low-dose well-ventilated-normalized ventilation (LDWV) distributions. The ventilation change was calculated in various dose regions based on the treatment plans using the DIR-registered before and after treatment data sets. The ventilation change based on TV-normalized ventilation was compared with the AV as well as the data normalized by PV and LDWV.

**Results:**

AV change may be misleading when the TV differs before and after treatment, which was found to be up to 6.7%. All three normalization methods produced a similar trend in ventilation change: the higher the dose to a region of lung, the greater the degradation in ventilation. In low dose regions (<5 Gy), ventilation appears relatively improved after treatment due to the relative nature of the normalized ventilation. However, the LDWV may not be reliable when the ventilation in the low-dose regions varies. PV exhibited a similar ventilation change trend compared to the TV-normalized in all cases. However, by definition, the ventilation distribution in the PV is significantly different from the original distribution.

**Conclusion:**

Normalizing ventilation distributions by the TV is a simple and reliable method for evaluation of ventilation changes.

## Introduction

Pulmonary ventilation *P* is defined as the fractional lung volume change during respiration [1] and can potentially be used as a surrogate for lung function change after the course of radiation therapy. Ventilation distribution matrix can be derived from four-dimensional CT (4D-CT) images using deformable image registration (DIR) [2-4]. Promising results have been reported on the ventilation data correlation between the 4D-CT and Xenon-enhanced CT [5]. The latter measures regional ventilation by observing the contrast gas, Xenon, wash-in or wash-out rate on serial CT images and is considered the gold standard for regional ventilation imaging [6]. However, Xenon gas is expensive and this modality is still technically challenging. Thus it is not commonly used clinically.

Currently, pulmonary ventilation imaging is mostly done using nuclear medicine techniques [7,8]. One of the advantages of ventilation data derived from 4D-CT, over nuclear imaging, is that they are quantitative. The values of ventilation for each voxel in the 4D-CT are determined by the volume changes between the end expiration and end inspiration phases of the 4D-CT. The volume changes can be calculated based on the Hounsfield unit (HU) change [2], by Jacobian of the deformation matrix [3], or directly (geometrically) using the deformation information [4]. 

Studies have been proposed to compare ventilation changes due to radiation treatment [9,10]. For such studies, at least two sets of 4D-CT are needed, one before and one after the radiation treatment.

The ventilation data derived from 4D-CT are based on the volume or Hounsfield unit (HU) number change between the end expiration and end inspiration phases. As a result, the volume or HU change depends on how deep the patient breathes when the 4D-CT is taken. The ventilation change would be misleading if the tidal volume (TV) changes between the two scans. One way to make the ventilation data comparable between different data sets is to change the ventilation distribution to a relative percentile distribution [11]. The idea is similar to the cumulative dose-volume histogram. If a certain percentage lung volume is covered by a certain ventilation value and below, this ventilation value is converted to the corresponding percentage value of the lung volume in the percentile distribution. Percentile distribution is appropriate for comparing ventilation derived from different image modalities, such as 4D-CT and single photon emission tomography (SPECT), due to the semi-quantitative nature of the SPECT ventilation data. However, as will be shown later, this method considerably alters the ventilation distribution shape. 

Another method is normalizing ventilation to the value of the low-dose well-ventilated volume [10]. The ventilation in the low-dose and well-ventilated (LDWV) regions should be stable throughout the treatment. In the study by Vinogradskiy et al. [10], the low-dose region was set at dose < 5 Gy and the well-ventilated region was set at ventilation > 50%. This method works well if there is good reproducibility of the ventilation distribution in the low dose region. However, as pointed out by Vinogradskiy et al., there were some ventilation variations in the low dose region in weekly images, potentially making this normalization method unreliable. 

This paper introduces a simple method that normalizes ventilation data from 4D-CT to the TV. This is a straightforward method for comparing two ventilation volumes because, by definition, there is a linear correlation between the ventilation value and TV. A similar normalization method using the average Jacobian values inside lungs was applied by Du et al. [12], in which the Jacobian method was employed in the ventilation calculation.

## Methods

### Deformable image registration (DIR) and patient data

The use of the patient data in this study followed a Moffitt Cancer Center’s Scientific Review Committee (SRC) and University of South Florida Institutional Review Board (IRB) approved protocol. Consent form was waived since this was a retrospective study using de-identified images of patients who have completed radiation therapy.

There are many different DIR algorithms used in research and clinical applications [13-21]. In this study diffeomorphic morphons (DM) [13] was used to generate deformation matrices. This DIR method was recently shown to be the most accurate one among a number of algorithms [22]. In its validation study, the average target registration error (TRE) for normal end-expiration-to-end-inspiration registration with one standard deviation (SD) was 1.4± 0.6 mm [22].

The deformation matrices were calculated between the end inspiration phase (0%) and end expiration phase (50%) of the 4D-CT for 6 lung cancer patients who had pre- and post-radiation treatment 4D-CT scans. Each of the patients was treated with stereotactic body radiation therapy (SBRT) to 50 Gy in 5 fractions. The deformation matrices were used to calculate the ventilation distributions. 

### Ventilation calculation

**Figure 1:**
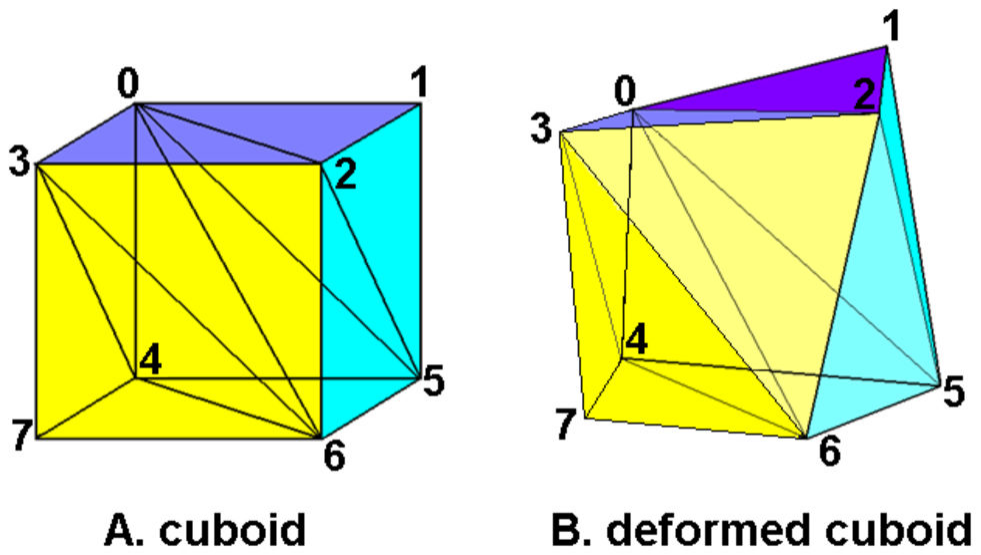
Any hexahedron can be divided into 6 tetrahedrons. (A) A cuboid can be divided into 6 tetrahedrons. (B) The corresponding deformed cuboid is composed of 6 deformed tetrahedrons.

The direct geometric algorithm, called ΔV method [4,23], was used for the ventilation calculations. Each CT voxel can be represented by a cuboid. The 8 vertices that compose the cuboid are changed to create a 12-face polyhedron. The polyhedron is still comprised of the 8 corresponding vertices. Any hexahedron can be divided into 6 tetrahedrons (Figure 1). The volumes of the cuboid and the deformed cuboid are the sums of the volumes of their 6 corresponding tetrahedrons. DIR establishes the correspondence between these vertices. In the local volume change calculation step, the volume of each voxel is calculated using the corresponding vertices of each respective polyhedron. 

The fundamental volume calculation is based on the volume calculation for each tetrahedron. The coordinates of the 4 vertices of a tetrahedron are used to determine its volume:

V=(b−a)⋅[(c−a)×(d−a)]/6 (1)

where ***a***, ***b***, ***c***, ***d*** are the vertices’ coordinates expressed as vectors. The volumes of the six tetrahedrons are summed up to generate the volume of the given polyhedron.

Pulmonary ventilation *P* is defined as the fractional volume change in respiration [1]: 

P=ΔV/V (2)

where *V* is the local volume at expiration and *ΔV* is the volume change from expiration to inspiration.

Considering two CT image sets, one taken at normal end expiration and the other taken at normal end inspiration, the volume of each voxel at expiration is determined simply by the CT voxel size. The voxels then expand during inspiration. The boundary of each voxel in the expiration image set is deformed. Deformable image registration determines the new boundaries. The volume calculation program calculates the volume of the deformed voxel. The volume change *ΔV* is the volume difference between the deformed voxel at inspiration and the original voxel at expiration.

### Tidal volume calculation and ventilation normalization

Tidal volume is calculated by integrating the local volume change *ΔV* over the entire lung volume. For two 4D-CT sets, taken before and after treatment, there are two TVs from the ventilation calculations: TV1 from the pre-treatment data set and TV2 from the post-treatment one. In the normalization process, the pre-treatment ventilation distribution is not changed, while the post-treatment ventilation distribution is normalized to TV1 by applying a multiplication factor, TV1/TV2, to every voxel in the lungs. After this normalization, both ventilation data sets have the same tidal volume, TV1, thus removing the final result dependency on the TV. In this study, two sets of ventilation data were compared using this normalization method for each patient.

The LDWV normalization method was used for comparison. The average ventilation value in the dose < 5 Gy and ventilation > 50% regions in the lungs was used to normalize the ventilation distribution for each data set. For the post-treatment data sets, in order to accurately use the dose in the < 5 Gy regions, the ventilation distribution was mapped to the pre-treatment CT using DIR and the normalization factor was then calculated based on the mapped distribution. 

The absolute ventilation (AV) data derived from DIR and 4D-CT were also converted to the relative percentile ventilation (PV) distribution. Thus four sets of ventilation distributions were generated for each patient for the ventilation change study: AV, LDWV- normalized, PV and TV-normalized. The ventilation change data were calculated in 4 dose regions based on the treatment plans: <5 Gy, between 5 and 20 Gy, between 20 and 30 Gy, and >30 Gy. 

## Results

**Figure 2:**
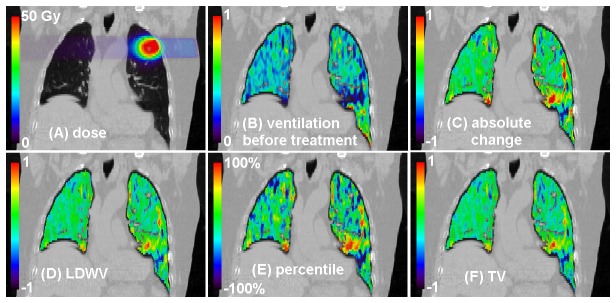
A coronal view of a typical case of ventilation change. Dose distribution is displayed in (A) and the absolute ventilation distribution before treatment is shown in (B). Absolute, LDWV normalized, percentile and TV normalized ventilation change distributions are shown in (C), (D) (E) and (F) respectively.

Figure 2 shows an example of the dose, ventilation and ventilation change distributions. More negative ventilation change, or decreased ventilation, can be seen in the high dose region in this case. The ventilation change distributions (lower row in Figure 2) were calculated using different normalization methods. The difference between the distributions of LDWV and TV normalization methods is not distinct without quantitative analysis. 

**Figure 3:**
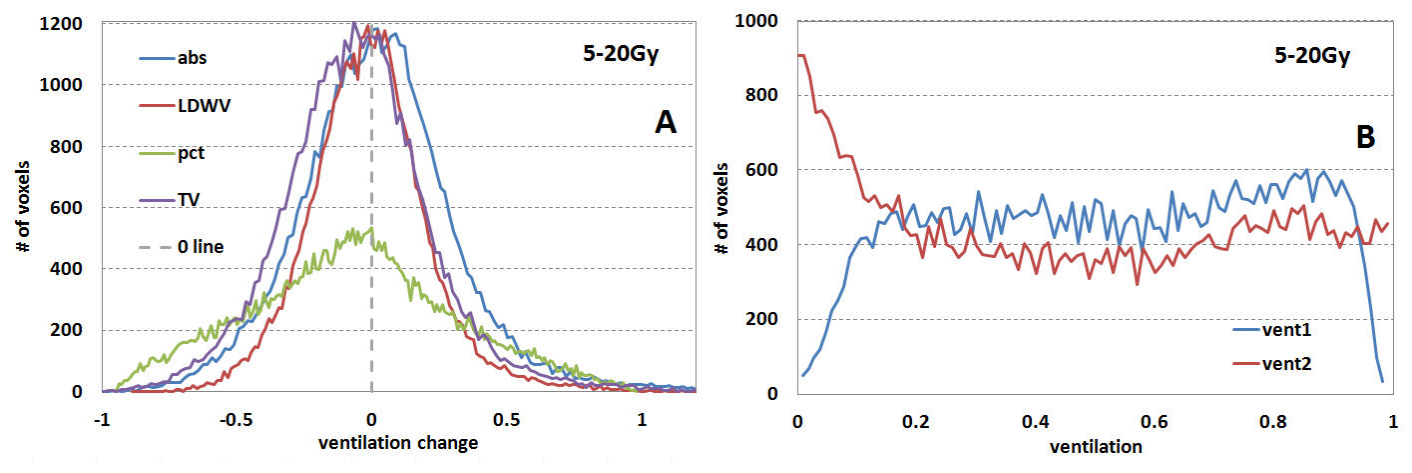
Ventilation and ventilation change distributions in the volume receiving 5 to 20 Gy for a typical case using different ventilation datasets. (A) Ventilation change distributions. Abs stands for absolute, pct for percentile, LDWV for low-dose well-ventilated normalized, TV for TV-normalized. The vertical dash line represents the value of no change. (B) Percentage ventilation distribution for the same case. vent1 stands for the pre-treatment ventilation, vent2 for the post-treatment ventilation.

Figure 3A shows the ventilation changes within the volume receiving 5 to 20 Gy for a patient using different ventilation datasets. The pre- and post-treatment PV data are also shown. By definition, the PV distribution is flat between 0 and 1 for the total lungs, very different from the other 3 sets of ventilation data. In this 5 to 20 Gy dose region, the PV distribution was not exactly flat, but the flat distribution can be observed, especially for the pre-treatment data (Figure 3B). By comparing two PV distributions, we conclude that in this case, most of the positive ventilation change came from the low ventilation region while the negative change is associated with the high ventilation area (Figure 3B).

The post-treatment ventilation data were mapped to the pre-treatment data set by DIR between the corresponding phases of the two 4D-CT datasets. The tumor volume was excluded from the analysis. As expected, the shapes of the distributions are about the same for the absolute, LDWV- and TV-normalized data sets, since the difference between the three data sets is only the normalization value, while the one for the percentile data set is different. For this patient, the TV in the second 4D-CT was 6.7% larger than in the first one. The AV change curve is shifted in the positive direction compared to TV- and LDWV-normalized curves. Based solely on the AV change data, ventilation appears to improve after treatment. This is obviously an artifact caused by the slightly larger TV in the after-treatment 4D-CT scan. After the TV normalization, the ventilation change is mostly negative (worse after treatment). 

**Figure 4:**
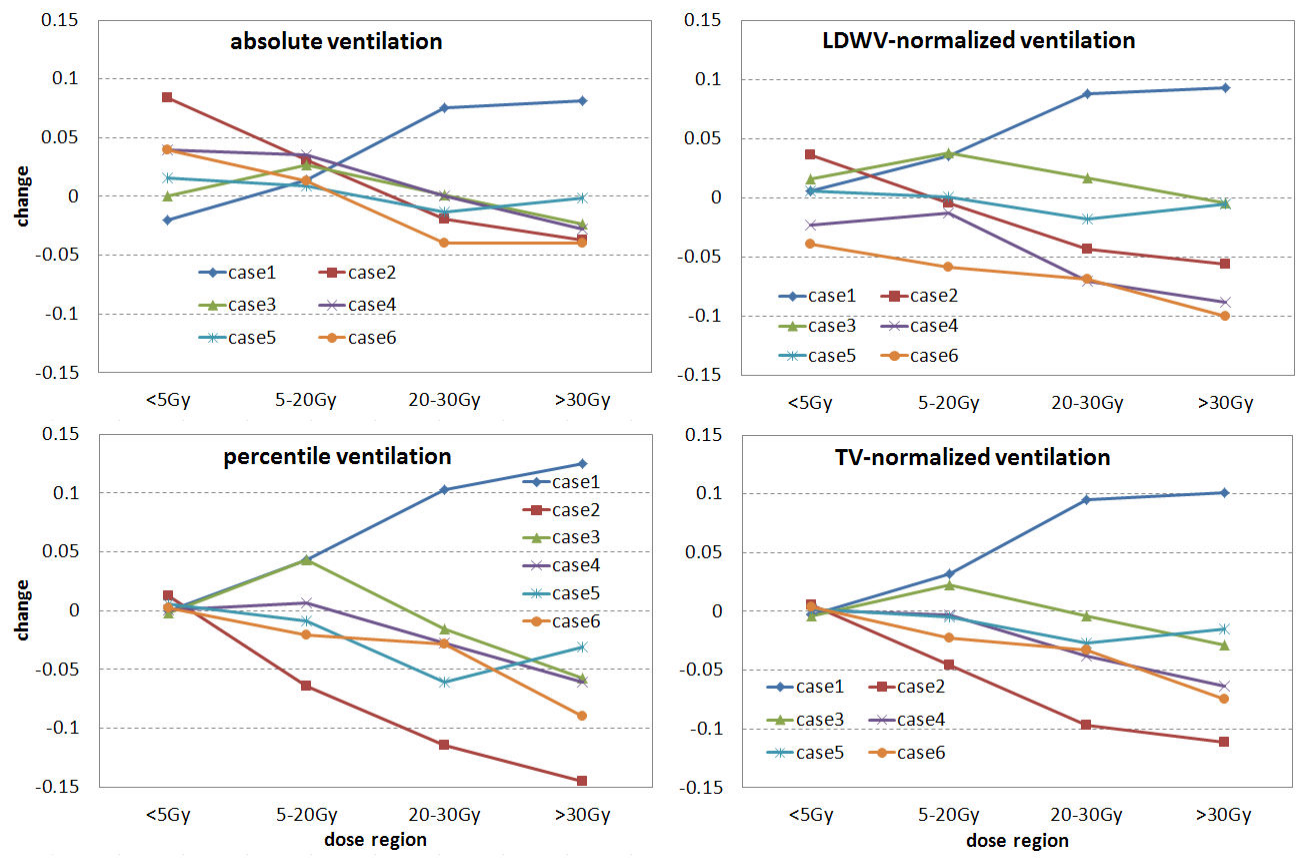
Ventilation change versus dose region curves for the 4 different ventilation data sets for all the 6 cases studied. LDWV stands for low-dose well-ventilated and TV for tidal volume.

Figure 4 shows the ventilation change versus dose region curves for the 6 studied cases with 4 different ventilation data sets. Only one out of the six cases showed increasing ventilation change (improving) with increasing dose (case1). The other 5 cases showed worsening ventilation with increased dose. In low dose regions (<5 Gy), ventilation appears relatively improved after treatment due to the relative nature of the normalized ventilation. Notice that the dose region under 5 Gy is the largest one while the over 30 Gy region is the smallest, usually with a two orders of magnitude volume difference between them. This is why in the percentile and TV-normalized ventilation figures a mostly small ventilation change in the <5 Gy region balances out the larger changes in other 3 dose regions. Table 1 lists the ventilation change versus dose region values for the 4 ventilation data sets.

**Table 1:** Ventilation change versus dose region.

Data set	Absolute	LDWV	Percentile	TV
<5Gy Median	0.028	0.006	0.001	0.002
<5Gy Range	-0.020−0.084	-0.039−0.036	-0.002−0.013	-0.004−0.005
5-20Gy Median	0.021	-0.002	-0.001	-0.004
5-20Gy Range	0.009−0.035	-0.059−0.038	-0.064−0.043	-0.046−0.032
20-30Gy Median	-0.006	-0.031	-0.028	-0.030
20-30Gy Range	-0.040−0.076	-0.070−0.088	-0.115−0.102	-0.097−0.095
>30Gy Median	-0.026	-0.031	-0.059	-0.046
>30Gy Range	-0.040−0.081	-0.100−0.093	-0.145−0.125	-0.111−0.101

In Figure 4, a negative ventilation change was found in all dose regions for two cases (case 4 and 6) for the LDWV-normalized ventilation. The reason for this violation of the relative nature of the normalized data was the inconsistence of the normalization factor used in the LDWV normalization. Table 2 lists the normalization factor variation between the LDWV and TV methods for the 6 cases. In the table, F1/F2 is the normalization factor ratio of the 1^st^ ventilation data (before treatment) to the 2^nd^ (after treatment) used in the LDWV normalization method, while TV1/TV2 is the TV ratio of the corresponding two data set. The % difference in the table was calculated as (F1/F2 - TV1/TV2)×100/(TV1/TV2). In theory, F1/F2 should follow TV1/TV2. For case 4 and 6, the F1/F2 value was smaller than TV1/TV2 value by 4.5% and 4.7% respectively. As a result, the post treatment ventilation data were normalized to a relatively large value, which caused “degraded” ventilation in all dose regions.

**Table 2:** Normalization factor variation between LDWV and TV normalization methods.

Case	1	2	3	4	5	6
LDWV F1/F2	1.026	0.968	1.017	0.923	0.992	0.923
TV1/TV2	1.016	0.933	0.995	0.967	0.987	0.968
% difference	0.97	3.77	2.19	-4.52	0.47	-4.72

A Dice similarity coefficient, DSC(A, B) = 2×|A∩B|/(|A|+|B|), was calculated between the volumes on which the LDWV normalization factor was based for the pre- and post-treatment ventilation data sets. The median value was 0.574 with the range of 0.466 to 0.591. This indicates that the ventilation in the low dose region is not very stable.

The TV-normalized ventilation change data closely follow the PV data (Figure 4). The AV data slightly overestimated the ventilation change due to the larger TV in the post-treatment 4D-CT scans (5 out of 6 cases, Table 2). The LDWV-normalized ventilation data were not reliable when the ventilation in the low-dose regions varied.

## Discussion

Originally, the TV-normalization method divides the ventilation values by the TV, which is numerically large, as it is obtained by integrating the local volume change ΔV over the entire lung. As a result, the TV-normalized ventilation values are very small numbers, which makes the presentation and interpretation of the results difficult. By renormalizing the ventilation data to the TV of the first data set, the numerical values are restored. Furthermore, ventilation data from sequential 4D-CT scans can be compared directly since the ventilation distribution no longer depends on the TV. Although the PV distribution is also independent of the TV, the shape of that distribution is substantially different (flat ventilation-volume distributions as shown in Figure 3B). 

The TV calculation using the deformation matrix is straightforward and easy to implement in the ventilation calculation programs. Even if other ventilation calculation algorithms are used, it is straight forward to calculate the tidal volume based on the ventilation distribution. Thus the TV normalization method can be applied when other ventilation calculation algorithms are used in this kind of studies. The calculation of the TV normalized ventilation is also quicker than the conversion to PV distribution. 

The normalization method proposed in this paper is similar to the one used by Du et al. [12]. When the Jacobian method is applied, the average Jacobian value over the lung is a straightforward quantity obtained from the calculation. Thus the ratio of the average Jacobian values can be naturally used for normalization. However, the ΔV approach is the most direct way of quantifying ventilation according to the definition. If this method is chosen, the TV is the quantity determined by the calculation and the ratio of the TV values can be easily used for normalization, while the Jacobian is not readily available.

Because of the inconsistency in the average ventilation in the LDWV region, the LDWV-normalized ventilation is generally not reliable and is not recommended. 

The goal of this paper was to introduce the TV normalization method for a ventilation change study of the ventilation data acquired at different times. A large cohort of patients is being analyzed in an ongoing study at our institution.

## Conclusions

An effective way of removing the tidal volume dependence of the ventilation data derived from 4D-CT is normalizing the absolute pre- and post-treatment ventilation data to the same tidal volume. Compared to the other normalization methods, the TV-normalized ventilation data consistently removes the tidal volume dependence and follows the absolute ventilation distribution closely, and therefore it should be a useful tool in ventilation change studies for patients undergoing radiotherapy.
